# Environments For Healthy Living (EFHL) Griffith birth cohort study: characteristics of sample and profile of antenatal exposures

**DOI:** 10.1186/1471-2458-12-1080

**Published:** 2012-12-15

**Authors:** Cate M Cameron, Paul A Scuffham, Rania Shibl, ShuKay Ng, Rani Scott, Anneliese Spinks, Gabor Mihala, Andrew Wilson, Elizabeth Kendall, Neil Sipe, Roderick J McClure

**Affiliations:** 1 School of Medicine, Griffith University, Nathan, QLD, 4131, Australia; 2 Griffith Health Institute, Griffith University, Nathan, QLD, 4222, Australia; 3 Commonwealth Scientific and Industrial Research Organisation (CSIRO) Ecosystem Sciences, Dutton Park, QLD, 4102, Australia; 4 Faculty of Health, Queensland University of Technology, Kelvin Grove, QLD, 4059, Australia; 5 School of Human Services and Social Work, Griffith University, Meadowbrook, Queensland, 4131, Australia; 6 School of Environment, Griffith University, Nathan, QLD, 4111, Australia; 7 Injury Research Institute, Monash University, Melbourne, Victoria, 3800, Australia

**Keywords:** Birth cohort, Longitudinal study, Epidemiology, Demographics, Descriptive analysis

## Abstract

**Background:**

The Environments for Healthy Living (EFHL) study is a repeated sample, longitudinal birth cohort in South East Queensland, Australia. We describe the sample characteristics and profile of maternal, household, and antenatal exposures. Variation and data stability over recruitment years were examined.

**Methods:**

Four months each year from 2006, pregnant women were recruited to EFHL at routine antenatal visits on or after 24 weeks gestation, from three public maternity hospitals. Participating mothers completed a baseline questionnaire on individual, familial, social and community exposure factors. Perinatal data were extracted from hospital birth records. Descriptive statistics and measures of association were calculated comparing the EFHL birth sample with regional and national reference populations. Data stability of antenatal exposure factors was assessed across five recruitment years (2006–2010 inclusive) using the Gamma statistic for ordinal data and chi-squared for nominal data.

**Results:**

Across five recruitment years 2,879 pregnant women were recruited which resulted in 2904 live births with 29 sets of twins. EFHL has a lower representation of early gestational babies, fewer still births and a lower percentage of low birth weight babies, when compared to regional data. The majority of women (65%) took a multivitamin supplement during pregnancy, 47% consumed alcohol, and 26% reported having smoked cigarettes. There were no differences in rates of a range of antenatal exposures across five years of recruitment, with the exception of increasing maternal pre-pregnancy weight (p=0.0349), decreasing rates of high maternal distress (p=0.0191) and decreasing alcohol consumption (p<0.0001).

**Conclusions:**

The study sample is broadly representative of births in the region and almost all factors showed data stability over time. This study, with repeated sampling of birth cohorts over multiple years, has the potential to make important contributions to population health through evaluating longitudinal follow-up and within cohort temporal effects.

**Trial registration:**

Australian and New Zealand Clinical Trials Registry ACTRN12610000931077

## Background

Health is the product of a complex interaction of factors relating to societal norms, a person’s local physical and social environment, and their biological and psychological capacities [1-3]. While public health researchers and practitioners recognise the importance of a multi-level, ecological model of disease causation and health promotion, [4,5] there has been limited research successfully quantifying these relationships in way that can support population based interventions [6]. The primary reason for this is that most previous aetiological and implementation research designs are not consistent with the multi-level logic that underpins our understanding of disease causation.

In order to advance the ecological understanding of disease causation, epidemiological research needs to be able to quantify the contextual factors as well as the proximal risk factors responsible for relevant health outcomes. There is a growing interest throughout the world in developing study designs that enable this level of multi-level analyses [7].

Environments for Healthy Living (EFHL) is a birth cohort study being conducted in South East Queensland, Australia, designed specifically to operationalise the ecological model of disease causation [5] and thus provide information required to develop policy driven improvements in population health. Using methods previously described, [8] data collected in this study include biological samples, participant surveys, medical record and administrative data linkage, land use and spatial variables. With a planned total cohort of nearly 4000 mother/infant dyads being recruited from a circumscribed study region, comprising communities across a broad range of socioeconomic exposures, this study has the capacity to address questions of contemporary importance.

The aim of this paper is to provide a comprehensive baseline profile for the first five recruitment years of the EFHL birth cohort and determine the ability to analyse the repeated cohort samples separately or combined. The study sample characteristics and maternal, household and, community exposures are described. Variation and data stability over recruitment years is examined.

## Methods

### Study design

EFHL is a repeated sample, longitudinal birth cohort in South East Queensland and Tweed in Northern New South Wales (NSW) Australia. The study has been registered with the Australian New Zealand Clinical Trials Registry (ACTRN12610000931077).

### Study population and participants

The study population includes all births from three geographically defined contiguous Health Districts (Logan, Beaudesert and the Gold Coast in Queensland; and Northern Rivers/Tweed in NSW) from 2006–2012. These districts cover an area of almost 6,000 square kilometres, encompassing approximately 30% of Queensland’s population [9]. Eligible participants were recruited from the three public maternity hospitals in the participating districts (Logan, Gold Coast and The Tweed Hospitals). Women attending private maternity hospitals, birthing centres and planned home births in the study region were not included. Women waiting for antenatal clinic appointments, on or after their routine 24 week antenatal visit, were approached by research trained midwives, provided with a detailed explanation of the study aims, and invited to participate. Pregnant women aged less than 16 years or unable to provide informed consent were excluded. The study sample is the offspring of women enrolled in EFHL.

### Recruitment

Women were recruited to participate in the EFHL study during four months of each year since 2006. As a consequence of unavoidable logistics involved with recruiting from multiple hospital sites, there was a small variation in the month recruitment began across the years. The pilot year commenced in November 2006; open recruitment from August in 2007 and 2008; and from 2009 onwards the four month recruitment period began in July. The EFHL cohort methodology has been described in full elsewhere [8]. Baseline data is currently available for five of the observational cohorts enrolled (2006–2010 inclusive).

### Data sources, instruments and scales

Baseline data was obtained from two key data sources namely a participant maternal baseline survey and hospital perinatal data related to the birth of the child. The maternal baseline questionnaire was self-administered, and consisted of 48 multi-item questions taking approximately 30 minutes to complete. Items included maternal, family and household characteristics, socio-economic factors, Kessler 6 (K6) psychological distress scale, [10,11] short form of the Family Environment Scale (FES), [12,13] neighbourhood and community connectedness (NCC), [14] maternal smoking and drinking behaviour, health supplement usage, and recreational substances used during pregnancy. Perinatal data was extracted from the medical records following maternal discharge from hospital. Data items included previous pregnancies, maternal conditions, obstetric care and complications, delivery information, and baby information such as gender, plurality, gestational age, birth weight and any complications. Information collected on the biological father has been specified as ‘paternal’ whereas information collected on the current partner (which was not necessarily the biological father) has been specified as ‘partner’ information.

Variable calculation and classifications include: Using Australian National birth weights for full-term singletons, birth weight was classified as low (<2500 g), normal (2500-4000 g) and high (>4000 g) [15]. BMI was calculated from weight in kilograms and height in meters (Weight/Height^2^). Pre-pregnancy maternal BMI measures were determined by self-report and paternal BMI measures by proxy-report from the maternal participant. BMI was classified as underweight (<18.5), normal weight (18.5-24.9), overweight (25.0-29.9) and obese (>30) [16]. Maternal and paternal ages were calculated in years at the time of the birth of their child.

Women and their partners were classified as employed if they reported working full-time, part-time, were self-employed or on paid maternity/paternity leave at the time of enrolment. Gross annual household income was reported in $10,000 increments and standardised in AUD$2010 values using published Consumer Price Index [17,18]. Standardised median household income is reported along with the income share in each quintile as an indicator of income distribution for the five recruitment years.

The K6 is intended to yield a global measure of “psychological distress” based on questions about the level of anxiety and depressive symptoms in the most recent 4-week period. The K6 has been widely used and has demonstrated excellent internal consistency, reliability and the ability to discriminate between community mental health cases and non-cases [10,19]. The six-items form a 24-point scale and the following cut-offs were used: No or low distress (0–7), moderate distress (8–12), and high distress (13–24) [11,20].

The NCC is a five item measure of perception of satisfaction with the local community [14]. NCC scores were classified as good (5–8), average (9–14) and poor (15–25) based on ±1 SD mean. Data was not available from the 2006 pilot cohort for the K6 and NCC.

For the purpose of this paper, self-reported alcohol consumption, recreational drug use, and multivitamin supplement use during pregnancy were dichotomised. Alcohol consumption was defined as any consumption of alcohol during pregnancy regardless of period, frequency, or quantity. Drug use for recreational or non-medical purposes, was asked using questions modified for pregnancy from the Australian National Drug Strategy Household Survey 2004 [21]. This included a range of drugs including steroids, barbiturates, cannabis, heroin, methamphetamines/amphetamines, cocaine, ecstasy, ketamine, solvents, and kava. A ‘prefer not to answer’ option was also provided. Multivitamin supplement use included any general multivitamin or pregnancy specific supplement taken during pregnancy.

### Analysis

Data cleaning and scoring was undertaken using SAS 9.2 software. Aggregate results were compared to regional and national reference population data from the Queensland and NSW Perinatal Data Collection systems, [22,23]. Australian Institute of Health and Welfare [24-27] and Australian Bureau of Statistics [28]. Changes in maternal, paternal, and household antenatal exposures were examined across each recruitment year to assess data stability. Measures of association and tests for trends were performed using Pearson’s chi-squared for nominal data and Gamma statistic for collapsed ordinal data. Differences in household incomes between the five years were tested with the Median-test [29]. Analyses were undertaken using SAS 9.2 and Stata 12. A significance level of 5% was used.

### Ethical approval

The protocol for the EFHL study was approved by the Griffith University Human Research Ethics Committee (MED/16/06/HREC, MED/23/11/HREC). Additional ethical approval for participant recruitment was also obtained from each of the three participating public maternity hospitals (Logan Hospital HREC/06/QPAH/96, Gold Coast Hospital HREC/06/GCH/52, The Tweed Hospital NCAHS HREC 358N). The protocol for the EFHL study conforms to the provisions of the Declaration of Helsinki in 1995 (as revised in Tokyo 2004). Each participant gave written informed consent for the release of hospital perinatal data related to the birth of their child, completion of a participant maternal baseline survey, and for individual follow-up. All research data are de-identified and stored for analysis.

## Results

### EFHL recruitment

**Figure 1:**
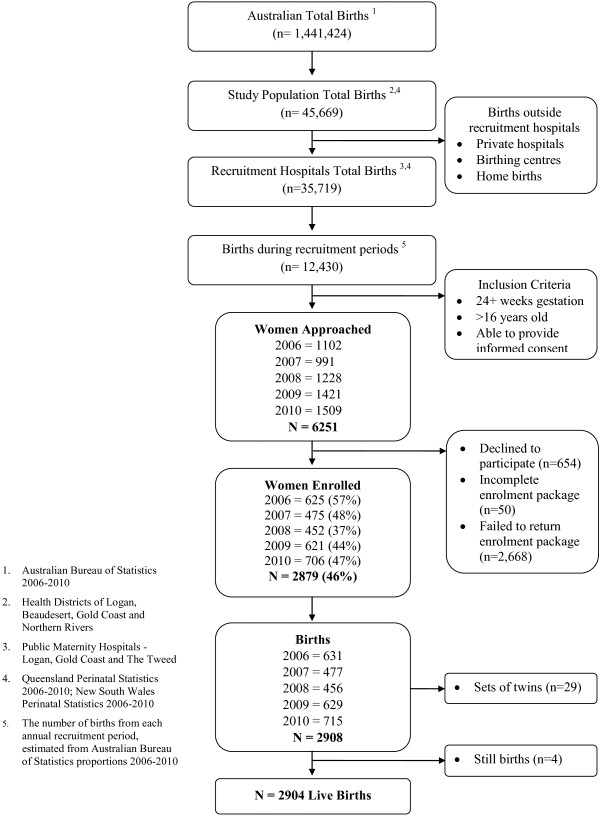
Flow diagram of eligibility and recruitment for the EFHL cohorts 2006–2010.

Figure 1 presents a flow diagram of the recruitment process, including all births in the Australian reference population and the study population (Logan, Beaudesert, Gold Coast and Northern Rivers Health Districts) from 2006 to 2010 inclusive [22,23]. An estimated 12,430 births occurred at the three recruitment hospitals during the annual four month recruitment periods between 2006 and 2010.

Of the total number of women approached (n=6,251), almost half (46%) were enrolled in the EFHL birth cohort study, with an average of 576 women enrolled each four-month recruitment period. A small number of women were not enrolled due to incomplete or missing questionnaires/consents which were unable to be resolved (n=50). The remaining either formally declined to participate when approached (n=654) or failed to return the enrolment packages (n=2,668). From 2006 to 2010 inclusive (five observational cohorts) 2,879 pregnant women were recruited, which resulted in 2904 live births with 29 sets of twins. EFHL participants accounted for approximately 6.4% of all births in the study population from the three Health Districts for the full five years (January 2006 – December 2010). The EFHL study sample represents 23.4% of the total births in the target population hospitals during the annual recruitment periods.

### EFHL infant sample characteristics

Table 1 presents the sample characteristics of the 2006–2010 EFHL birth cohorts with comparative regional study and national reference population data. There were some differences in the birth characteristics of the EFHL sample when compared to population data. EFHL has a lower representation of early gestational babies with 3.3% of EFHL babies born less than 37 weeks gestation, versus 7.0% regionally and 8.2% nationally (p<0.001). The study sample has fewer still births, and a lower proportion of babies weighing less than 2500 g. There were no differences in the gender distribution or the plurality of births from expected study population rates.

**Table 1:** Sample characteristics of the EFHL infant cohorts 2006–2010, compared with regional and national population data

	**EFHL study sample**	**Study population**^**a**^	**Reference population**^**b**^	**P-value**
**Infant Characteristics**	**N=2908**	**N=45,669**	**N=1,441,424**	
**Gender of infant**				
Males	1448 (50.5)	23,498 (51.5)	735,479 (51.4)	0.315 (Study)
Females	1420 (49.5)	22,170 (48.5)	694,495 (48.6)	0.312 (Reference)
Missing	40	1	11,450	
**Plurality**				
Singleton	2850 (98.0)	44,568 (97.6)	1,395,372 (96.8)	0.173 (Study)
Multiple	58 (2.0)	1,101 (2.4)	46,052 (3.2)	<0.001 (Reference)
Missing	0	0	0	
**Gestational age at birth**				
<28 weeks	0 (0.0)	273 (0.6)	12,881 (0.9)	<0.001 (Study)
28-36 weeks	95 (3.3)	2,912 (6.4)	105,235 (7.3)	<0.001 (Reference)
37-41 weeks	2734 (96.1)	42,227 (92.5)	1,308,933 (90.8)	
>41 weeks	17 (0.6)	245 (0.5)	13,761 (1.0)	
Missing	62	12	614	
**Live births**				
Live	2904 (99.9)	45,368 (99.3)	1,430,500 (99.2)	0.001 (Study)
Still born	4 (0.1)	301 (0.7)	10,815 (0.8)	<0.001 (Reference)
Missing	0	0	109	
**Birth weight**				
<2500g	76 (2.7)	2,484 (5.4)	97,524 (6.8)	<0.001 (Study)
2500-3999g	2319 (81.6)	37,287 (81.7)	1,170,968 (81.3)	<0.001 (Reference)
>4000	448 (15.7)	5,894 (12.9)	172,169 (11.9)	
Missing	65	4	763	

^**a**^ Study population includes all births in the Logan, Beaudesert, Gold Coast and Northern Rivers Health Districts. Data sources: Queensland Perinatal Statistics 2006–2010; New South Wales Perinatal Statistics 2006–2010.

^b^ Reference Population includes all births in Australia 2006–2010. Data sources: National Perinatal Statistics 2006–2010.

^c^ Pearsons chi-squared.

### Profile of maternal, household and demographic antenatal exposures

The maternal age of women in the EFHL study ranged from 16 years to 48 years, with a median age of 29 years. The EFHL cohort was younger when compared with all women giving birth in the study region, with more women aged 20–24 years (p=0.001) [24-27]. The paternal age ranged from 15 years to 70 years, with a median age of 31 years. The proportion of high risk pregnancy complications, including gestational diabetes (5.3%) and essential hypertension (<1%) were consistent with Queensland rates [24-27].

Table 2 presents the antenatal exposure data collected for each of the five cohort years (2006–2010). The study sample has a higher proportion of mothers born outside of Australia compared with national maternal population data (28.8% versus 25.4%) [24-27]. Over 35% of the mothers in the study were either overweight or obese with pre-pregnancy BMIs >25. Of note, less than half of all mothers had a pre-pregnancy BMI in the normal weight range. More than half of the fathers were reported to be either overweight or obese (53.0%). Of the women enrolled, almost 21% did not complete high school education and almost half were either unemployed or not in the labour force at the time of enrolment. This included those who were on disability pensions and homemakers. Similarly, 22.7% of partners did not complete high school education and 7.5% were unemployed or not in the labour force at the time of enrolment. The majority of families were reported as having two parents in the household and 38.9% reported having no other children living in the house at the time of baseline enrolment (Table 2).

**Table 2:** Profile of maternal, family and household antenatal exposures for the EFHL cohorts 2006-2010

	**2006 (n=625)**	**2007 (n=475)**	**2008 (n=452)**	**2009 (n=621)**	**2010 (n=706)**	**All Years (n=2879)**	**P value**
**Maternal Characteristics**	n (%)	n (%)	n (%)	n (%)	n (%)	n (%)	
**Maternal age**							
<25 years	147 (23.6)	118 (24.9)	112 (24.8)	142 (22.9)	184 (26.1)	**703 (24.5)**	0.5367^a^
25-29 years	172 (27.5)	126 (26.5)	123 (27.3)	199 (32.2)	195 (27.7)	**815 (28.4)**	
30-34 years	187 (30.0)	133 (28.0)	119 (26.4)	142 (22.9)	180 (25.6)	**761 (26.5)**	
35-39 years	100 (16.0)	76 (16.0)	77 (17.1)	105 (17.0)	114 (16.2)	**472 (16.4)**	
40+ years	18 (2.9)	22 (4.6)	20 (4.4)	31 (5.0)	31 (4.4)	**122 (4.2)**	
Missing = 6							
**Maternal BMI**							
Underweight (<18.5)	104 (16.8)	80 (17.1)	77 (17.2)	109 (17.7)	120 (17.2)	**490 (17.2)**	0.0349^a^
Normal weight (18.5-24.9)	320 (51.8)	225 (48.1)	222 (49.5)	278 (45.3)	311 (44.6)	**1356 (47.7)**	
Overweight (25.0-29.9)	111 (18.0)	89 (19.0)	89 (19.9)	119 (19.4)	137 (19.7)	**545 (19.1)**	
Obese (>30)	83 (13.4)	74 (15.8)	60 (13.4)	108 (17.6)	129 (18.5)	**454 (16.0)**	
Missing = 34							
**Country of Birth**							
Australia	460 (73.6)	348 (73.3)	313 (69.4)	434 (69.9)	493 (69.9)	**2048 (71.2)**	0.3392^b^
Other	165 (26.4)	127 (26.7)	138 (30.6)	187 (30.1)	212 (30.1)	**829 (28.8)**	
Missing = 2							
**Highest level of education**							
Not complete school	129 (20.7)	93 (19.7)	88 (19.6)	147 (23.8)	143 (20.4)	**600 (20.9)**	0.4967^a^
Completed high school	201 (32.3)	163 (34.5)	139 (30.9)	178 (28.8)	212 (30.2)	**893 (31.2)**	
Trade/Apprenticeship	179 (28.7)	131 (27.7)	135 (30.0)	186 (30.1)	198 (28.3)	**829 (28.9)**	
University degree	114 (18.3)	86 (18.2)	88 (19.6)	107 (17.3)	148 (21.1)	**543 (19.0)**	
Missing = 14							
**Employment Status**							
Employed	314 (50.4)	241 (51.2)	223 (49.8)	310 (50.5)	355 (50.6)	**1443 (50.5)**	0.2176^b^
Unemployed	47 (7.5)	48 (10.2)	47 (10.5)	68 (11.1)	88 (12.5)	**298(10.4)**	
Not in labour force	262 (42.1)	182 (38.6)	178 (39.7)	236 (38.4)	259 (36.9)	**1117(39.1)**	
Missing = 21							
**Paternal / Partner Characteristics**^**d**^							
**Paternal age**							
<25 years	100 (16.2)	72 (15.4)	81 (18.1)	91 (14.9)	123 (17.6)	**467 (16.4)**	0.7330^a^
25-29 years	169 (27.3)	113 (24.2)	123 (27.5)	175 (28.6)	182 (26.0)	**762 (26.8)**	
30-34 years	160 (25.9)	144 (30.8)	114 (25.5)	156 (25.5)	186 (26.6)	**760 (26.7)**	
35-39 years	120 (19.4)	92 (19.7)	84 (18.8)	121 (19.7)	131 (18.7)	**548 (19.3)**	
40+ years	69 (11.2)	46 (9.9)	45 (10.1)	70 (11.4)	77 (11.0)	**307 (10.8)**	
Missing = 35							
**Paternal BMI**							
Underweight (<18.5)	127 (20.6)	87 (18.3)	86 (19.2)	125 (20.5)	129 (18.6)	**554 (19.5)**	0.3110^a^
Normal weight (18.5-24.9)	169 (27.4)	125 (26.4)	127 (28.4)	171 (28.0)	192 (27.6)	**784 (27.5)**	
Overweight (25.0-29.9)	229 (37.1)	178 (37.6)	171 (38.3)	206 (33.7)	239 (34.4)	**1023 (36.0)**	
Obese (>30)	92 (14.9)	84 (17.7)	63 (14.1)	109 (17.8)	135 (19.4)	**483 (17.0)**	
Missing = 35							
**Partners highest level of education**							
Not complete school	128 (22.0)	88 (20.0)	106 (24.8)	139 (24.3)	148 (22.3)	**609 (22.7)**	0.9906^a^
Completed high school	153 (26.3)	126 (28.6)	96 (22.4)	146 (25.5)	170 (25.6)	**691 (25.7)**	
Trade/Apprenticeship	227 (39.1)	173 (39.3)	174 (40.7)	230 (40.1)	247 (37.3)	**1051 (39.1)**	
University degree	73 (12.6)	53 (12.1)	52 (12.1)	58 (10.1)	98 (14.8)	**334 (12.4)**	
Missing = 194							
**Partners employment status**							
Employed	552 (93.7)	420 (93.7)	395 (93.0)	535 (91.3)	612 (91.2)	**2514 (92.5)**	0.2499^b^
Unemployed	22 (3.7)	11 (2.5)	15 (3.5)	33 (5.6)	33 (4.9)	**114 (4.2)**	
Not in labour force	15 (2.6)	17 (3.8)	15 (3.5)	18 (3.1)	26 (3.9)	**91 (3.3)**	
Missing =160							
**Household Characteristics**							
**Annual household income**^**e**^							
Median	$62,051	$63,295	$62,866	$62,733	$63,786	**$62,946**	0.4301^c^
Lowest quintile	7.4%	6.8%	7.0%	6.6%	7.0%	**7.0%**	
2nd quintile	13.2%	13.5%	12.3%	12.9%	12.5%	**12.9%**	
3rd quintile	17.9%	17.6%	16.9%	17.8%	16.5%	**17.3%**	
4th quintile	23.1%	23.1%	22.9%	22.8%	22.2%	**22.8%**	
Highest quintile	38.3%	39.0%	40.9%	40.0%	41.8%	**40.0%**	
Missing = 449							
**Family status**							
Sole parent family	89 (14.4)	65 (13.7)	57 (12.7)	83 (13.4)	81 (11.6)	**375 (13.1)**	0.6477^b^
Two parent family	531 (85.6)	409 (86.3)	393 (87.3)	535 (86.6)	616 (88.4)	**2484 (86.9)**	
Missing = 20							
**Children in household**							
No children	284 (46.3)	169 (36.7)	163 (36.6)	214 (34.5)	279 (39.5)	**1109 (38.9)**	0.0138^a^
1-3 children	310 (50.6)	275 (59.7)	265 (59.4)	380 (61.2)	398 (56.4)	**1628 (57.2)**	
4 or more children	19 (3.1)	17 (3.7)	18 (4.0)	27 (4.4)	29 (4.1)	**110 (3.9)**	
Missing = 32							
**Changed place of residence in past year**							
Moved	282 (45.2)	212 (44.8)	192 (43.4)	257 (42.3)	288 (41.9)	**1231 (43.4)**	0.7118^b^
Did not move	342 (54.8)	261 (55.2)	250 (56.6)	351 (57.7)	399 (58.1)	**1603 (56.6)**	
Missing = 45							
**Satisfaction with area of residence**							
Satisfied	542 (86.9)	414 (87.3)	390 (87.3)	526 (85.2)	605 (86.6)	**2477 (86.6)**	0.6536^a^
Neither	57 (9.1)	41 (8.7)	44 (9.8)	72 (11.7)	72 (10.3)	**286 (10.0)**	
Dissatisfied	25 (4.0)	19 (4.0)	13 (2.9)	19 (3.1)	22 (3.1)	**98 (3.4)**	
Missing = 18							
**Perceived neighbourhood community connectedness**							
Strong	NA	59 (12.7)	65 (14.6)	110 (18.1)	101 (14.7)	**335 (15.2)**	0.1297^a^
Average	NA	314 (67.8)	299 (67.3)	383 (63.1)	475 (69.0)	**1471 (66.8)**	
Poor	NA	90 (19.4)	80 (18.0)	114 (18.8)	112 (16.3)	**396 (18.0)**	
Missing = 677							
**Maternal Health and Behaviours during pregnancy**							
**Maternal psychological distress**							
Low distress	NA	363 (77.6)	346 (78.6)	478 (79.8)	568 (82.7)	**1755 (80.0)**	0.0191^a^
Moderate distress	NA	81 (17.3)	77 (17.5)	98 (16.4)	98 (14.3)	**354 (16.1)**	
High distress	NA	24 (5.1)	17 (3.9)	23 (3.8)	21 (3.1)	**85 (3.9)**	
Missing = 685							
**Cigarette smoking**							
Smoked	159 (25.5)	127 (26.9)	117 (25.9)	167 (27.1)	179 (25.4)	**749 (26.1)**	0.9466^b^
Did not smoke	465 (74.5)	346 (73.1)	335 (74.1)	450 (72.9)	525 (74.6)	**2121 (73.9)**	
Missing = 9							
**Alcohol use**							
Consumed alcohol	316 (50.7)	252 (53.2)	223 (49.3)	277 (44.9)	287 (40.8)	**1355 (47.2)**	<0.0001^b^
No alcohol consumed	307 (49.3)	222 (46.8)	229 (50.7)	340 (55.1)	417 (59.2)	**1515 (52.8)**	
Missing = 9							
**Recreational drug use**							
Used drugs	45 (7.4)	33 (7.0)	29 (6.6)	34 (5.6)	34 (4.9)	**175 (6.2)**	0.3526^b^
Did not use drugs	566 (92.6)	440 (93.0)	411 (93.4)	574 (94.4)	659 (95.1)	**2650 (93.8)**	
Missing = 54							
**Multivitamin supplement use**							
Used supplements	NA	305 (66.9)	273 (61.2)	407 (67.4)	445 (64.1)	**1430 (65.0)**	0.1529^b^
Did not use supplements	NA	151 (33.1)	173 (38.8)	197 (32.6)	249 (35.9)	**770 (35.0)**	
Missing = 679							

^a^ Gamma statistic for ordinal data,^b^ Pearsons chi-squared test for nominal data,^c^ Median-test for ordinal data,^d^‘Paternal’ indicates biological father, ‘Partner’ indicates current relationship partner not necessarily the biological father, ^e^Gross annual household incomes standardised in AUD$2010 values.

At the time of enrolment the median total gross household income was $62,946 in AUD$2010 values; the lowest income quintile contributed 7.0% to the total wealth of the cohort whereas the highest income quintile contributed 40.0%. The EFHL cohort demonstrated lower household incomes when compared with Queensland household incomes for 2006–2010 [17]. In Queensland, the lowest income quintile contributed 4.2% and the highest income quintile contributed 45.7% to the total wealth.

While the majority of families reported satisfaction with the area in which they lived (86.6%), only 15.2% reported a strong level of neighbourhood and community connectedness, with 43.4% of families having moved in the 12 months prior to enrolment. Almost two thirds of women reported taking either a multi-vitamin or pregnancy supplement during the pregnancy. Over a quarter of the women reported smoking cigarettes during pregnancy, 47.2% reported having consumed alcohol, and 6.2% of women reported some form of recreational drug use during the pregnancy (Table 2). Cannabis was the most common recreational drug used during pregnancy (74.9%), with ecstasy, methamphetamines/amphetamines and heroin combined accounting for a further 23.4%. Almost 4% of women scored Kessler 6 high psychological distress levels and 16.1% moderate distress levels in the four weeks prior to enrolment.

### Data stability of antenatal exposures measured for each recruitment cohort

The five cohorts (2006–2010) were examined for temporal differences in rates of twenty different exposure characteristics across the five years. For the majority of antenatal exposures, there were no significant differences across the years of recruitment, with rates of each factor remaining stable for the five calendar years (Table 2).

Of the four factors where a statistically significant change over time was detected, one showed an anomaly only in the 2006 pilot data. A higher proportion of families had no children living in the household when enrolled in 2006, compared to the four subsequent years of recruitment. When the 2006 pilot data were excluded, this factor remained stable and was no longer statistically significant.

The temporal analysis indicated an increase in the self-reported pre-pregnancy BMI of women being enrolled in the EFHL study between 2006 and 2010. While the proportion of women with a normal pre-pregnancy weight decreased over the five calendar years, the proportion of women with a pre-pregnancy BMI greater than 30 showed some increase, with 18.5% of the cohort self-reporting pre-pregnancy obesity in 2010 (p=0.0349).

Across the five years, the rates of reported maternal psychological distress changed. Fewer women scored high levels of baseline psychological distress, and conversely there was a significant increase across the calendar years in the proportion of women with low psychological distress scores during pregnancy (p=0.0191).

The factor that demonstrated the most notable variation over the five recruitment years was the self-reported consumption of alcohol during pregnancy. In 2006, 50.7% of women reported consumption of alcohol and this steadily declined over the five years with a 40.8% consumption of alcohol during pregnancy reported in 2010 (p<0.0001).

## Discussion

The results of the analyses of the baseline questionnaire and birth details provide a robust basis for developing a valid and useful epidemiological quantification of the ecological determinants of health. The large study sample is broadly representative of the majority of deliveries in the region, with sufficient range of exposure data to support on-going analyses of research questions relating to the social determinants of health. Temporal and cohort effect analyses indicate strong data stability across five recruitment years.

The three public maternity hospitals from which the study recruited, accounted for 78% of all births in the study population during 2006–2010. The remaining 22% of births in the study region occurred either at private hospitals, birthing centres, or were home births and were not represented in the EFHL study sample. The proportion of public hospital deliveries in the study population was higher than the national average of 70% [27]. Public hospitals are known to serve a disproportionately larger share of patients of relatively low socio-economic status [30]. Women giving birth in public hospitals have been found to be younger, a higher proportion are first births, a greater proportion smoke, and more women present with medical conditions such as hypertension and diabetes [31]. The Health Districts of the study region are known to have higher proportions of socio-economic disadvantage and more people with non-English speaking backgrounds than the national average [9]. These characteristics were similarly reflected in the EFHL sample who demonstrated lower incomes shares, younger age, more overseas born and high proportions of smoking than the national average.

The findings related to the birth sample characteristics suggested that while sampling was broadly representative of the target study population, there were some differences. The EFHL sample included births with greater gestational age, more singletons, higher birth weights, and fewer still births than that of the study population. This study sampled women who were of 24 weeks gestation or later, in waiting rooms attending their routine antenatal clinic visits at public hospitals, during the latter half of the calendar year (except in the 2006 pilot year). Not all antenatal clinics were able to be attended by the research midwifes and short patient waiting times in some clinics prevented the approach of all women, which is likely to have contributed to only half of the estimated births during the recruitment periods being approached for the study. In addition, routine antenatal clinic visits typically include women assessed as low risk births carrying singletons or twins, excluding higher multiple pregnancies or those women with known health conditions associated with an increased risk of birthing complications (mainly premature babies <28 weeks gestation). Two studies of South East Queensland births data have shown some seasonal differences in pre-term births, birth weight, and limb length in full-term singleton babies, with heavier babies born in the winter months which coincide with our recruitment periods [32,33].

The repeated sampling design was used in this study to enable the assessment and quantification of the impact of structural or environment changes and health policy implementation during the total recruitment period. The results of the analyses presented here in relation to changing prevalence of alcohol consumption during pregnancy suggest this repeated sampling design is a key strength of the study. Foetal alcohol syndrome and the consumption of alcohol during pregnancy have been the increasing focus of research nationally and internationally, [34-36] resulting in changes to national practice guidelines in 2009, [37] and the development of various state media campaigns in recent years targeting young women, alcohol, and pregnancy [38-40]. Australian studies have found approximately 50%-60% of women drink some alcohol during pregnancy [34,35,41]. However, there is a need for temporal examination of prevalence patterns of consumption to have insight into the effectiveness of policy dissemination [42]. The findings of this EFHL study present the first repeated prevalence measure of alcohol consumption during pregnancy in the same geographic population over a five year period. The results demonstrated a 9.9% reduction in the consumption of alcohol during pregnancy from 2006 to 2010. While beyond the scope of this paper, further analysis will be conducted with regards to changes in risk groups, periods of consumption, frequency, quantity and binge drinking.

The stability of the repeated sample recruitment process can be assessed by comparing the baseline characteristics and antenatal exposure data for each annual cohort. Few of the socio-demographic baseline characteristics of the repeated samples showed significant differences over the five years of data examined. Pre-pregnancy BMI increased over the five calendar years reflecting trend changes found in the national population for the same time period [43]. Caution must be taken with a few individual exposures that demonstrated change over time and a single factor that showed instability in the pilot year when data collection methods were being established (number of children living in the household). However, these maternal and socio-demographic factors are not seasonal, and are therefore unlikely to be affected by differences in the months of recruitment. The overall results of this analysis indicate strong robustness of the data and a high degree of stability.

## Conclusion

This paper presents a comprehensive baseline profile of the first five recruitment years of the EFHL birth cohort. Stability of data ascertainment across the recruitment years is strong. The broad representation of socio-economic status, community measures and key proximal exposures such as tobacco, alcohol, and drug intake during pregnancy provides the range of exposure required to ensure identification of effect for relevant risk factors as study outcomes become prevalent. This study, with multiple cohorts from repeated sampling, has the potential to assess health policy implementation during the study period and make important contributions to population health.

## Abbreviations

BMI: Body Mass Index; CPI: Consumer Price Index; EFH: Environments for Healthy Living; FES: Family Environment Scale; K6: Kessler 6; NCC: Neighbourhood and Community Connectedness; NSW: New South Wales.

## Competing interests

The authors declare that they have no competing interests.

## Authors’ contributions

RJM and CMC conceived the idea of EFHL. CMC drafted the manuscript. All authors contributed to revisions and have read and approved the final manuscript.

## Pre-publication history

The pre-publication history for this paper can be accessed here:

http://www.biomedcentral.com/1471-2458/12/1080/prepub
